# Pneumococcal Vaccination Uptake Among People with HIV Following the Implementation of an On-Site Vaccination Service in an Italian Specialist Out-Patient Clinic

**DOI:** 10.3390/vaccines14020176

**Published:** 2026-02-13

**Authors:** Anna Lisa Ridolfo, Maria Vittoria Cossu, Letizia Oreni, Cristina Gervasoni, Giacomo Casalini, Debora Visigalli, Catia Rosanna Borriello, Andrea Giacomelli, Andrea Gori, Spinello Antinori

**Affiliations:** 1 III Division of Infectious Diseases, ASST Fatebenefratelli Sacco, 20157 Milan, Italy; marialetizia.oreni@gmail.com (L.O.); cristina.gervasoni@asst-fbf-sacco.it (C.G.); casalini.giacomo@asst-fbf-sacco.it (G.C.); andrea.giacomelli@unimi.it (A.G.); spinello.antinori@unimi.it (S.A.); 2 II Division of Infectious Diseases, ASST Fatebenefratelli Sacco, 20157 Milan, Italy; maria.cossu@asst-fbf-sacco.it (M.V.C.); andrea.gori@unimi.it (A.G.); 3 Pharmaceutical Department, ASST Fatebenefratelli Sacco, 20157 Milan, Italy; visigalli.debora@asst-fbf-sacco.it; 4 Prevention Department, ASST Fatebenefratelli Sacco, 20157 Milan, Italy; catia.borriello@asst-fbf-sacco.it; 5 Department of Biomedical and Clinical Sciences, Università Degli Studi di Milano, 20157 Milan, Italy

**Keywords:** pneumococcal disease, pneumococcal vaccine, HIV, vaccine hesitancy, immigrants, Italy

## Abstract

**Background:** People with HIV (PWH) face elevated risk of pneumococcal disease despite optimal antiretroviral therapy. In Italy, pneumococcal vaccination coverage in adults remains suboptimal. For PWH, access barriers may be amplified because adult vaccinations are primarily delivered in primary care, whereas HIV care is mostly hospital-based. **Methods:** We conducted a retrospective, observational study at an HIV clinic in Milan, Italy, evaluating the impact of an on-site vaccination service implemented in January 2019. At baseline, 1854 PWH were in active follow-up; 135 (7.3%) had previously received pneumococcal vaccination. We assessed vaccination uptake (PCV13-PPSV23 sequential schedule or PCV20) among the remaining 1719 unvaccinated individuals through December 2023. A logistic regression analysis was used to identify factors associated with vaccine acceptance. **Results:** Over five years, 745/1719 individuals (43.3%) either initiated PCV13 + PPSV23 or received PCV20, representing a six-fold increase from baseline. Of 639 individuals receiving PCV13, 80.1% completed the sequence with PPSV23. Most vaccinations (80.8%) were administered on-site. In multivariable analysis, men who have sex with men showed higher uptake (aOR 1.56, 95% CI 1.25–1.95), while regular and irregular immigrants had significantly lower uptake (aOR 0.70 and 0.24, respectively) compared to Italian nationals. **Conclusions:** Integration of vaccination services into routine HIV care substantially improved pneumococcal vaccination uptake. However, with nearly half of eligible patients remaining unvaccinated, additional strategies are required to address vaccine hesitancy and inequities, particularly among immigrants, to achieve optimal pneumococcal coverage in PWH.

## 1. Introduction

Pneumococcal disease (PD), including pneumococcal pneumonia and invasive pneumococcal disease (IPD), remains a leading preventable cause of morbidity and mortality in adults, particularly among older individuals and those with chronic or immunocompromising conditions, including human immunodeficiency virus (HIV) infection [1,2,3]. People with HIV (PWH) are at substantially elevated risk: a recent population-based study from the Netherlands reported seven- and eight-fold higher rates of IPD and pneumococcal pneumonia, respectively, compared with the general population, with risk remaining four- to five-fold higher even among those optimally treated with combination antiretroviral therapy (cART) [4,5].

Pneumococcal vaccines have been shown to be safe and immunogenic in PWH, although immune responses may be reduced compared to immunocompetent individuals [6]. Considering the substantial disease burden and benefits of vaccination, pneumococcal immunization has long been recommended for PWH by most international guidelines [7,8]. However, vaccination uptake in this population remains suboptimal in many countries [9,10,11,12]. In Italy, pneumococcal vaccination using the sequential schedule with 13-valent pneumococcal conjugate vaccine (PCV13) followed by 23-valent polysaccharide pneumococcal vaccine (PPSV23) has been recommended and offered free of charge to adults ≥65 years and high-risk individuals, including PWH, since the 2017–2019 National Immunization Prevention Plan (Piano Nazionale di Prevenzione Vaccinale, PNPV), which had set a coverage target of 75% [13]. The PNPV 2023–2025 reinforced these recommendations [14], and many Italian Regions have begun implementing protocols favoring a single dose of the newly approved PCV20. However, the target has not been achieved: a 2022 nationwide survey found only 39.5% vaccination coverage among eligible adults, with limited awareness and perceived access difficulties identified as key barriers [15].

An estimated 150,000 people live with HIV in Italy (prevalence: 0.3 per 100 residents) [16], yet data on pneumococcal vaccination uptake in this population remain limited. For PWH, access barriers may be amplified by the structural fragmentation of healthcare services. Although HIV specialists in hospital-based clinics are highly knowledgeable about vaccination recommendations for PWH and their specific immunization needs, adult vaccination services in Italy are traditionally organized and delivered outside specialty clinics, primarily through general practitioners and public vaccination centers. This separation may result in missed vaccination opportunities, particularly given that many PWH have limited engagement with primary care or may be reluctant to disclose their HIV status in non-specialized settings because of stigma [17]. Integrating vaccination into hospital-based HIV care may help to reduce organizational barriers and improve access by leveraging established relationships between PWH and their HIV specialists. However, evidence supporting the effectiveness of this strategy is limited.

This study evaluated the impact of implementing a hospital-based vaccination service on pneumococcal vaccination uptake among PWH attending a specialized HIV clinic in Italy, comparing uptake before and after the service introduction and identifying factors associated with vaccine acceptance.

## 2. Materials and Methods

### 2.1. Study Design and Setting

This was a retrospective, observational, single-center study conducted at the outpatient HIV clinic of the Infectious Diseases Unit III, Luigi Sacco Hospital, Milan, Italy.

Since 1st January 2019 (index date), an on-site vaccination service has been offered to all PWH in active follow-up at our clinic, in collaboration with the local Prevention Department and the Hospital Pharmacy. The service operated three days per week, with 3.5 h sessions, and was staffed by four HIV specialists and four nurses working in shifts, all trained in vaccine administration, vaccination planning, vaccine procurement, and storage.

HIV specialists had access to the regional immunization registry (Sistema Informativo Anagrafe Vaccinale Regionale, SIAVR) through a dedicated electronic portal, allowing review of patients’ complete immunization histories. During routine clinic visits, HIV specialists could use this information to assess immunization status, provide vaccine counseling, and issue vaccination recommendations when deemed appropriate. Vaccination appointments were scheduled using a dedicated electronic agenda and, whenever possible, coordinated with routine HIV follow-up visits to minimize additional patient visits. Vaccines were administered by trained nurses under physician supervision in a dedicated clinic room.

In addition to pneumococcal vaccination, the service actively offered vaccines against hepatitis A and B, human papillomavirus, meningococcus B and ACWY, *Haemophilus influenzae* type B, tetanus, varicella, herpes zoster, and measles–mumps–rubella.

When multiple vaccines were indicated, the administration plan was individualized based on the patient’s clinical characteristics and risk profile, without a predefined prioritization hierarchy. Pneumococcal vaccines were co-administered with other vaccines whenever clinically appropriate, and in the absence of contraindications, in accordance with the Centers for Disease Control and Prevention (CDC) guidelines [18].

The present study focused exclusively on pneumococcal vaccination uptake because it has a clearly defined, guideline-recommended schedule for all PWH, with discrete and measurable completion endpoints that remain stable over time, making them suitable for a longitudinal cohort analysis. Other recommended vaccines with different schedules and completion endpoints (such as influenza and COVID-19 vaccines) were not included in the present analysis.

According to the Italian PNPV and regional health policies, pneumococcal vaccination was actively offered free of charge to all PWH regardless of citizenship. During the early study period, a sequential schedule with the 13-valent pneumococcal conjugate vaccine (PCV13), followed by the 23-valent pneumococcal polysaccharide vaccine (PPSV23) after 6–12 months, was used. From May 2022 onward, previously unvaccinated individuals received a single dose of the 20-valent pneumococcal conjugate vaccine (PCV20), in accordance with regional protocols.

### 2.2. Service Interruption

The vaccination service was temporarily interrupted between March and May 2020 due to the COVID-19 pandemic.

### 2.3. Study Population

This study was conducted on a cohort of PWH aged ≥ 18 years who were in active follow-up at the index date (1 January 2019). Active follow-up was defined as having had at least one clinical contact with the center in the 12 months preceding the index date and being alive at the index date.

Pneumococcal vaccination status for the entire cohort was assessed at the index date to estimate baseline vaccination uptake. For the longitudinal analysis evaluating the impact of the on-site vaccination service on vaccine uptake, we included 1719 individuals who had not previously received pneumococcal vaccination. Individuals who initiated care after the index date or who had prior pneumococcal vaccination were excluded. Patients were followed until the earliest of vaccination completion, death, or the end of the study period.

### 2.4. Data Collection

Demographic and clinical data were extracted from electronic clinical records and collected in a standardized electronic case report form (CRF). Variables included age, biological sex, citizenship (Italian, documented immigrant, undocumented immigrant), country and region of birth, occupation (employed, unemployed, student, retired, unknown), mode of HIV acquisition (men who have sex with men [MSM], heterosexual contact, injection drug use [IDU], other or unknown), date of HIV diagnosis, date of antiretroviral therapy (ART) initiation, previous AIDS-defining events, CD4+ T-cell count and HIV-RNA level closest to the index date, and comorbidities (cardiovascular disease, chronic kidney disease, diabetes mellitus, and oncological disease).

Dates of administration of PCV13, PCV20, and PPSV23 and place where the vaccines were administered were obtained from clinical charts and/or the SIAVR registry. Patients were followed until death, transfer to another clinical center, or the end of the study period. For individuals who transferred to other centers before completing pneumococcal vaccination, vaccination and vital status were ascertained through the SIAVR registry (which includes vital status information) or, when necessary, by telephone contact.

### 2.5. Outcome of Interest

The primary outcome was uptake of pneumococcal vaccination, defined as initiation of the PCV13–PPSV23 sequential schedule or receipt of a single dose of PCV20. Secondary outcome was the rate of completion of the PCV13 + PPS23 vaccination schedule.

### 2.6. Statistical Analyses

The proportion of individuals who had received any pneumococcal vaccine by the index date was calculated for the entire cohort (baseline uptake). Among those unvaccinated at baseline, vaccination uptake over time was evaluated, and cumulative uptake was calculated by calendar year. Changes in vaccination status over time were assessed for statistical significance using McNemar’s test for paired data.

Baseline demographic and clinical characteristics of vaccinated (i.e., those who received at least PCV 13 or PCV 20) and unvaccinated subjects were compared using the χ^2^ test or Fisher’s exact test, as appropriate, for categorical variables, and the Wilcoxon rank-sum test for continuous variables.

A logistic regression model was used to evaluate factors associated with pneumococcal vaccination uptake during follow-up. Candidate variables (age ≤ 65 vs. >65 years, sex, citizenship, and mode of HIV acquisition) were selected a priori based on clinical and epidemiological relevance. Univariable logistic regression analyses were first performed for all candidate variables, and results are reported as odds ratios (ORs) with 95% confidence intervals (CIs).

A parsimonious multivariable modeling strategy was then adopted to minimize collinearity and overfitting while focusing on clinically meaningful determinants. Sex was excluded from the multivariable model because of strong correlation with mode of HIV acquisition, leaving three non-collinear variables in the final model. Adjusted odds ratios (aORs) with 95% CIs were calculated for variables included in the final model. In this model, multicollinearity was assessed using variance inflation factors (VIFs), which ranged from 1.01 to 1.02, indicating no evidence of collinearity, and model fit was evaluated using the Hosmer–Lemeshow goodness-of-fit test, which indicated adequate lack of fit (*p* > 0.05). All variables included in the final model had complete data, and no imputation was required.

All of the statistical analyses were conducted using SAS software, version 9.4, and differences with a *p* value of <0.05 were considered statistically significant.

### 2.7. Ethical Statement

The study was conducted in accordance with the principles of the Declaration of Helsinki and the International Conference on Harmonization and Good Clinical Practice Guidelines. The local Ethics Committee (Comitato Etico Interaziendale Area 1) approved the study procedures (approval number: 0034581/2023). All participants provided written informed consent.

## 3. Results

At the time of implementation of our on-site vaccination service (1st January 2019), 1854 PWH were in active follow-up at our clinic. Of these, 135 (7.3%) had previously received pneumococcal vaccination (PCV13 alone or PCV13 followed by PPSV23), representing the baseline pneumococcal vaccination uptake prior to the service activation. The remaining 1719 unvaccinated individuals constituted the primary study population for evaluating the impact of the on-site vaccination service on vaccine uptake.

**Figure 1:**
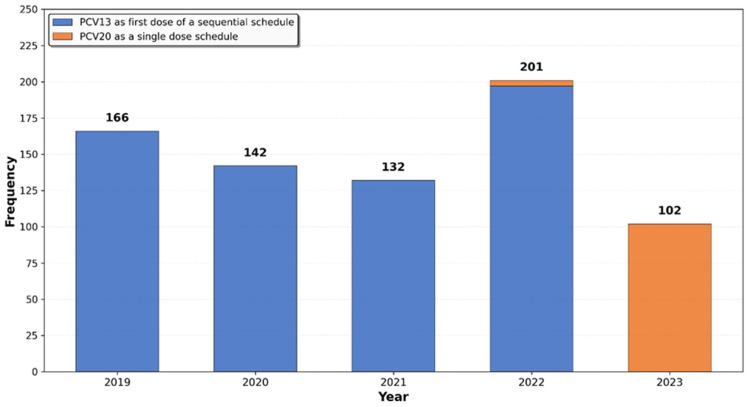
Number of patients receiving pneumococcal vaccine by year, 2019–2023.

Vaccination status was successfully ascertained for all 1719 eligible participants. Over the 5-year observation period, 745 (43.3%) of the 1719 initially unvaccinated individuals initiated the PCV13–PPSV23 sequential schedule or received a single dose of PCV20. A rapid increase in vaccination uptake was observed soon after the service activation, with a peak of 201 newly vaccinated individuals in 2022 (Figure 1).

**Figure 2:**
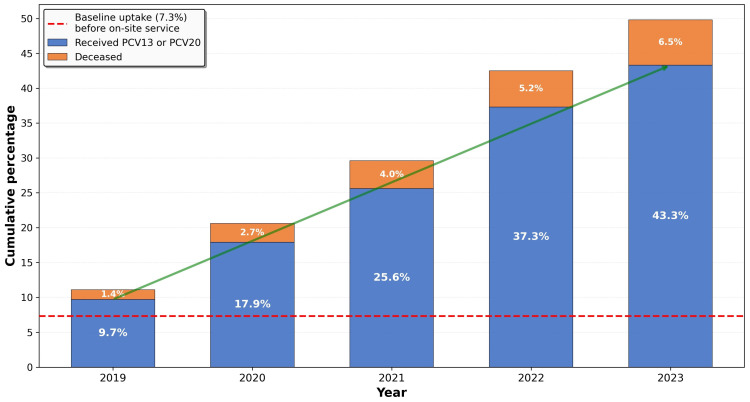
Cumulative rate of pneumococcal vaccine update over time, 2019–2023.

Figure 2 illustrates the progressive increase in cumulative vaccination uptake over the study period. From a baseline uptake of 7.3% before the on-site service implementation, vaccination uptake increased steadily to 43.3% by December 2023—a six-fold increase that was highly statistically significant (*p* < 0.001).

Of the 745 vaccinated individuals, 639 (85.8%) received PCV13 and 106 (14.2%) received PCV20, which became available from May 2022 onward. Of those who received PCV13, 512 (80.1%) completed the sequential schedule with PPSV23 during the study period. Overall, 618/745 vaccinated individuals (82.9%) completed their respective vaccination schedules with PCV13 + PPSV23 or PCV20.

Most vaccinations were administered through the in-clinic vaccination service (*n* = 602, 80.8%). The remaining vaccinations were administered by general practitioners (*n* = 37, 5.0%), public immunization services (*n* = 56, 7.5%), or at an unspecified site (*n* = 50, 6.7%).

Baseline characteristics of the study population and comparisons between vaccinated and unvaccinated individuals are reported in Table 1.

Vaccinated individuals were slightly older (median age 53 vs. 52 years; *p* = 0.014), and more frequently males (78.0% vs. 69.6%; *p* < 0.001) and Italians (87.5% vs. 81.6%; *p* < 0.001); among the 272 immigrant participants, the majority originated from sub-Saharan Africa (47.8%) or South America (27%). They were also more frequently retired (8.6% vs. 4.8%; *p* = 0.022). Mode of HIV acquisition differed significantly between groups, with vaccinated individuals more frequently reporting MSM as mode of HIV transmission and less frequently heterosexual transmission (37.4% vs. 29.1% and 40.9% vs. 47.7%, respectively; *p* = 0.003). No significant differences were observed in time since HIV diagnosis or history of AIDS-defining conditions. Although virologic suppression rates were high in both groups (>90%), vaccinated individuals less frequently had detectable HIV-RNA (2.3% vs. 7.5%; *p* < 0.001) and slightly higher median CD4+ counts (716 vs. 700 cells/μL; *p* = 0.021). Cardiovascular comorbidities were more prevalent among vaccinated individuals (23.2% vs. 16.6%; *p* = 0.001).

Table 2 presents the results of univariable and multivariable logistic regression analyses examining factors associated with vaccination uptake. In multivariable analysis, MSM transmission was independently associated with higher vaccination odds (aOR 1.56, 95% CI 1.25–1.95; *p* < 0.001), while immigrant status was independently associated with lower vaccination odds (aOR 0.70, 95% CI 0.52–0.94; *p* = 0.016, and aOR 0.24, 95% CI 0.11–0.56; *p* = 0.001 for regular and undocumented immigrants, respectively).

## 4. Discussion

Our study demonstrates that the implementation of a dedicated on-site vaccination service within an Italian HIV clinic was associated with a substantial improvement in pneumococcal vaccination uptake among PWH. At baseline, vaccination coverage was strikingly low, with only 7% of patients having received pneumococcal vaccination, underscoring the presence of significant access barriers in this population, despite long-standing guideline recommendations and free-of-charge access to vaccination.

Over the five-year observation period, 43.3% of previously unvaccinated individuals received pneumococcal vaccination with either PCV13 or PCV20, corresponding to a six-fold increase compared with the pre-intervention period. In addition, more than 80% of patients who initiated the PCV13 schedule completed the recommended sequential vaccination with PPSV23. This completion rate is considerably higher than those reported in other real-world settings, including immunocompromised populations in Germany [19] and an elderly cohort in Italy [20], where adherence has been consistently poor (4% and 2.3%, respectively).

The uptake of PCV20 was limited in our cohort, due to its relatively recent introduction during the latter part of the study period. However, the availability of PCV20 represents an important opportunity to further improve vaccination coverage among high-risk populations, including PWH. Beyond its broader serotype coverage and predicted higher impact on invasive pneumococcal disease, the simplified single-dose schedule may reduce logistical barriers, facilitate provider recommendation, and enhance patient adherence compared with sequential strategies [21]. These advantages are particularly relevant in populations requiring multiple preventive interventions.

The majority of vaccinations were administered directly within the HIV clinic, highlighting the feasibility and acceptability of integrating vaccination services into routine HIV care. This integrated model likely benefited from the strong therapeutic alliance between patients and HIV specialists, as well as from high levels of engagement in care, as evidenced by optimal immuno-virological status on cART in the vast majority of patients. for almost all of the patients.

Our findings are consistent with previous evidence showing that clinic-based vaccination programs tailored to specific patient populations can significantly increase vaccine uptake. Similar benefits have been reported among PWH [22] and other vulnerable groups, such as patients with cancer or inflammatory bowel disease, where on-site vaccination has been shown to reduce missed opportunities and improve adherence by leveraging established patient–provider relationships and minimizing logistical complexity [23,24,25].

Despite these improvements, nearly half of eligible patients remained unvaccinated by the end of the study period, indicating that removing structural barriers alone is insufficient to achieve optimal vaccination coverage. Vaccine hesitancy, broadly defined as delayed acceptance or refusal of vaccination despite availability, likely played a role [26]. Contributing factors may include limited knowledge about pneumococcal disease, concerns about adverse effects, doubts regarding vaccine effectiveness, fear of injections, and a low perceived personal risk, all of which may be influenced by psychological, cultural, and socio-economic determinants [26].

Notably, our analysis identified important disparities in vaccination uptake.

Although descriptive analyses showed higher vaccination uptake with increasing age, age >65 years was not significantly associated with pneumococcal vaccination in the multivariable model, despite older age being an independent risk factor for severe pneumococcal disease beyond HIV-related immunosuppression. This finding may reflect limited statistical power, given that only 8.7% of PWH were aged >65 years, as well as incomplete recognition of cumulative risk by both patients and healthcare providers, highlighting a potential target for educational interventions.

Immigrant status—particularly undocumented immigration—was independently associated with lower vaccination rates, despite free-of-charge access to recommended vaccinations being guaranteed by the national healthcare system regardless of citizenship. This finding aligns with previous studies highlighting the impact of language barriers, limited health literacy, cultural differences, and mistrust in healthcare systems on vaccination behaviors among immigrant populations [27,28]. In our study, the majority of immigrants originated from low-income regions of Africa and South America, which may further exacerbate these barriers due to limited prior access to healthcare and preventive services. These results emphasize the need for targeted and culturally sensitive interventions, such as multilingual educational materials and tailored counseling, to address persistent inequities in access to preventive care.

Conversely, MSM showed higher vaccination uptake, which is consistent with prior reports [29,30]. This pattern may reflect greater familiarity with preventive health interventions, given long-standing vaccination and screening programs targeting MSM, including those for human papillomavirus (HPV) and hepatitis A. Identifying and adapting successful strategies from this subgroup could help inform interventions aimed at improving vaccine acceptance in other populations.

Beyond patient-level factors, provider-related barriers must also be considered, as insufficient or ineffective communication by healthcare providers may represent a greater obstacle to vaccination than patient refusal [31]. A cross-sectional survey conducted among 338 PWH in the Paris region showed that the main reason for vaccine reluctance was the lack of vaccination proposals/reminders [11]. Consistently, a recent Italian survey among adults with chronic medical conditions reporting a pneumococcal vaccination rate of approximately 16%, showed that only 17% of participants had been advised by their primary care provider to receive the vaccine [32].

Although HIV specialists are well positioned to deliver accurate and personalized vaccine information, time constraints, competing clinical priorities, and limited training in addressing vaccine hesitancy may hinder effective discussions and provision of advice on recommended vaccinations. Addressing these challenges will require both targeted training in vaccine communication and organizational changes that allow sufficient time for meaningful preventive counseling within routine care [33].

Overall, our study supports the feasibility and added value of embedding vaccination services into the HIV care continuum. However, achieving optimal and equitable pneumococcal vaccination coverage will likely require a multifaceted approach that combines structural changes with patient- and provider-focused interventions. Reminder systems, standing orders, dedicated counseling visits, and enhanced coordination between hospital-based clinics, primary care providers, public immunization services, and community pharmacies may further reduce missed opportunities for vaccination [34]. This study has several limitations. First, its single-center, retrospective design may limit the generalizability of our findings. Our clinic serves a predominantly urban population in Northern Italy, and results might differ in rural settings or regions with different healthcare structures, patient demographics, or resource availability. Second, the absence of a concurrent control group precludes definitive causal attribution of the observed improvements solely to the intervention. Although the temporal association is strong, we cannot exclude the influence of secular trends, increased awareness of pneumococcal vaccination during the study period, or other concurrent initiatives. Third, the study period overlapped with the COVID-19 pandemic (2020–2023), which may have influenced vaccination behaviors in multiple ways [35]. While the pandemic led to temporary service interruptions (March–May 2020), it may also have heightened awareness of vaccine-preventable diseases and willingness to vaccinate; conversely, pandemic-related disruptions and vaccine fatigue may have negatively affected uptake in some individuals. Another limitation is the lack of complete data on the reasons why a substantial proportion of the study population remained unvaccinated during the study period. Future prospective studies should systematically assess factors influencing vaccine acceptance and hesitancy—including health literacy, language barriers, cultural beliefs, provider communication practices, and social influences—which are essential to design tailored interventions aimed at achieving optimal vaccination coverage and reducing disparities, particularly among more vulnerable PWH. Additionally, data on previous episodes of pneumonia or IPD were not systematically collected and could not be included in the analysis. This information might have provided additional insights into factors influencing vaccination uptake or clinical outcomes, although current guidelines recommend pneumococcal vaccination for all PWH regardless of prior infection history. Finally, our study focused exclusively on pneumococcal vaccination, and it remains unclear whether this integrated care model would be equally effective for other recommended vaccines in PWH. However, evidence suggests that vaccination behaviors may be interconnected. A large-scale study among individuals with chronic diseases demonstrated that patients who received pneumococcal vaccination had more than 15 times higher odds of also receiving influenza vaccination, indicating that acceptance of one vaccine predicts acceptance of others [36]. Therefore, while our findings are specific to pneumococcal vaccination, they may have broader implications for comprehensive vaccine uptake strategies in PWH, warranting further investigation into whether similar integrated care approaches could enhance adherence to complete vaccination schedules in this population.

Despite these limitations, our study has notable strengths, including a large cohort size, a five-year follow-up period, the use of a validated regional immunization registry to confirm vaccination status, and the evaluation of a real-world, scalable intervention implemented within a hospital-based specialist setting.

## 5. Conclusions

Our findings highlight several actionable strategies to improve pneumococcal vaccination uptake among PWH. Integrating vaccination delivery within HIV clinics can reduce access barriers and leverage established patient–provider relationships. Targeted outreach to immigrant populations, supported by multilingual and culturally sensitive counseling, may help address inequities in vaccine access and acceptance. Simplifying vaccination schedules by prioritizing single-dose vaccines, such as PCV20 or the newly available PCV21, could improve acceptance and completion rates. Systematic assessment of vaccination status through immunization registries, coupled with provider reminders, can further reduce missed opportunities. Overall, a multifaceted, equity-focused approach that combines structural integration with patient- and provider-level interventions appears essential to enhance vaccination coverage and address disparities in this vulnerable population.

## Figures and Tables

**Table 1:** Characteristics of the study population and differences observed between vaccinated and unvaccinated subjects.

	Total *n* = 1719	Vaccinated *n* = 745 (43.3%)	Not Vaccinated *n* = 974 (56.7%)	*p* Value
Age, median years (IQR)	52.0 (45.0–57.0)	53.0 (45.0–58.0)	52.0 (45.0–56.0)	0.014
Age > 65 years	150 (8.7)	70 (9.4)	80 (8.2)	0.39
Sex (%)				<0.001
Male	1259 (73.2)	581 (78.0)	678 (69.6)	
Female	460 (26.8)	164 (22.0)	296 (30.4)	
Mode of HIV acquisition, *n* (%)				0.003
HE	770 (44.8)	305 (40.9)	465 (47.7)	
MSM	562 (32.7)	279 (37.4)	283 (29.1)	
IDU	320 (18.6)	135 (18.1)	185 (19.0)	
Other	67 (3.9)	26 (3.5)	41 (4.2)	
Citizenship, *n* (%)				<0.001
Italian	1447 (84.2)	652 (87.5)	795 (81.6)	
Regular immigrant	236 (13.7)	86 (11.5)	150 (15.4)	
Irregular immigrant	36 (2.1)	7 (0.9)	29 (3.0)	
Employment status, *n* (%)				0.022
Employed	1368 (79.6)	586 (78.7)	782 (80.3)	
Unemployed	141 (8.2)	57 (7.7)	84 (8.6)	
Student	17 (1.0)	8 (1.1)	9 (0.9)	
Retired	111 (6.5)	64 (8.6)	47 (4.8)	
ND	82 (4.8)	30 (4.0)	52 (5.3)	
Previous AIDS, *n* (%)	400 (23.3)	169 (22.7)	231 (23.7)	0.645
Years from HIV diagnosis, median years (IQR)	17.4 (10.1–25.6)	17.5 (10.1–26.2)	17.4 (10.1–25.3)	0.620
Time on ART, median years (IQR)	13.9 (7.4–21.3)	14.3 (7.5–21.5)	13.6 (7.3–21.1)	0.18
Current CD4 cells/μL, median (IQR)	706 (534–905)	716 (560–929)	700 (520–885)	0.021
CD4 count < 200 cells/μL, *n* (%)	35 (2.0)	11 (1.5)	24 (2.5)	0.17
HIV-RNA > 50 copies/mL, *n* (%)	129 (7.5)	17 (2.3)	73 (7.5)	<0.001
Comorbidities, *n* (%)				
Diabetes	129 (7.5)	59 (7.9)	70 (7.2)	0.58
Cardiovascular disease	335 (19.5)	173 (23.2)	162 (16.6)	0.001
Renal disease	127 (7.4)	62 (8.3)	65 (6.7)	0.226
Cancer	205 (11.9)	85 (11.4)	120 (12.3)	0.599
Died during the study period, *n* (%)	111 (6.5)	19 (2.6)	92 (9.4)	<0.001

List of abbreviations: *n*, number; IQR, inter quartile range; HE, heterosexual; MSM, men who have sex with men; IDU, intravenous drug users; ND, not determined; ART, antiretroviral treatment.

**Table 2:** Uni-and multivariable logistic regression analysis of factors associated with pneumoccocal vaccine uptake.

	OR	OR 95% CI	*p*-Value	aOR	aOR 95% CI	*p*-Value
Age (years)						
≤65	1			1		
>65	1.16	0.83–1.62	0.390	0.579	0.78–1.56	0.579
Sex						
Males	1			-	-	
Females	0.65	0.52–0.81	<0.001	-	-	
HIV risk factor						
HE	1			1		
MSM	1.50	1.21–1.88	<0.001	1.56	1.25–1.95	<0.001
IDU	1.11	0.85–1.45	0.430	1.05	0.80–1.37	0.746
Other	0.97	0.58–1.61	0.897	0.93	0.56–1.56	0.792
Citizenship						
Italians	1			1		
Regular immigrants	0.70	0.53–0.93	0.014	0.70	0.52–0.93	0.016
Irregular immigrants	0.29	0.13–0.68	0.004	0.24	0.10–0.56	0.001

Variables included in the final multivariable model: age (>65 vs. ≤65 years), citizenship (Italian vs. documented immigrant and undocumented immigrant), and HIV mode of acquisition (heterosexual vs. MSM, IDU, other/unknown). List of abbreviations: OR, odds ratio; aOR, adjusted odds ratio; CI, confidence interval; HE, heterosexual; MSM, men who have sex with men; IDU, intravenous drug users.

## Data Availability

The data presented in this study are not publicly available due to privacy and ethical restrictions concerning sensitive patient information. Anonymized data are available from the corresponding author upon reasonable request and with appropriate institutional approval.
